# Ethyl 6-methyl­sulfanyl-2-phenyl-1*H*-imidazo[1,2-*b*]pyrazole-7-carboxyl­ate monohydrate

**DOI:** 10.1107/S1600536809010988

**Published:** 2009-03-31

**Authors:** Teng-fei Shao, Gui-long Zhao, Jian-wu Wang

**Affiliations:** a School of Chemistry and Chemical Engineering, Shandong University, Jinan 250100, People’s Republic of China

## Abstract

The title compound, C_15_H_15_N_3_O_2_S·H_2_O, has been obtained in a search for new imidazo[1,2-*b*]pyrazole derivatives with better biological activity. The 1*H*-imidazo[1,2-*b*]pyrazole plane forms a dihedral angle of 16.90 (3)° with the benzene ring. π–π inter­actions are indicated by the short distance of 3.643 (2) Å between the centroids of the benzene and imidazole rings. The crystal structure also involves inter­molecular O—H⋯N hydrogen bonds.

## Related literature

For the biological activity of imidazo[1,2-*b*]pyrazole derivatives, see: Vanotti *et al.* (1994 ▶); Kinnamon *et al.* (2000 ▶); Li *et al.* (2005 ▶). For bond-length data, see: Allen *et al.*, 1987 ▶.
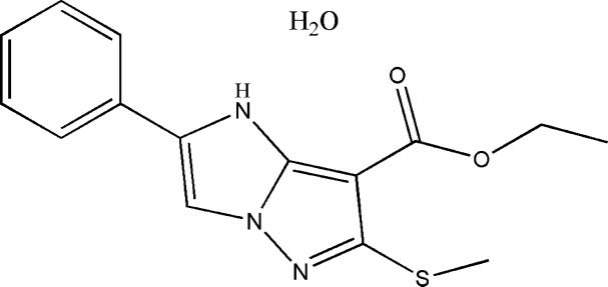


## Experimental

### 

#### Crystal data

C_15_H_15_N_3_O_2_S·H_2_O
*M* *_r_* = 319.38
Orthorhombic, 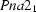

*a* = 19.017 (2) Å
*b* = 5.4854 (7) Å
*c* = 15.2314 (18) Å
*V* = 1588.9 (3) Å^3^
*Z* = 4
Mo *K*α radiation
μ = 0.22 mm^−1^
*T* = 273 K
0.20 × 0.10 × 0.10 mm

#### Data collection

Bruker SMART CCD area-detector diffractometer
Absorption correction: multi-scan (*SADABS*; Sheldrick, 1996 ▶) *T*_min_ = 0.957, *T*_max_ = 0.978
8763 measured reflections
3326 independent reflections
1877 reflections with *I* > 2σ(*I*)
*R*_int_ = 0.051

#### Refinement

*R*[*F* ^2^ > 2σ(*F* ^2^)] = 0.044
*wR*(*F* ^2^) = 0.094
*S* = 0.97
3326 reflections
207 parameters
1 restraint
H atoms treated by a mixture of independent and constrained refinement
Δρ_max_ = 0.15 e Å^−3^
Δρ_min_ = −0.14 e Å^−3^
Absolute structure: Flack (1983 ▶), 1442 Friedel pairs
Flack parameter: −0.01 (9)

### 

Data collection: *SMART* (Bruker, 1998 ▶); cell refinement: *SAINT* (Bruker, 1999 ▶); data reduction: *SAINT*; program(s) used to solve structure: *SHELXTL* (Sheldrick, 2008 ▶); program(s) used to refine structure: *SHELXTL*; molecular graphics: *SHELXTL*; software used to prepare material for publication: *SHELXTL*.

## Supplementary Material

Crystal structure: contains datablocks I, global. DOI: 10.1107/S1600536809010988/hg2495sup1.cif

Structure factors: contains datablocks I. DOI: 10.1107/S1600536809010988/hg2495Isup2.hkl

Additional supplementary materials:  crystallographic information; 3D view; checkCIF report

## Figures and Tables

**Table 1:** Hydrogen-bond geometry (Å, °)

*D*—H⋯*A*	*D*—H	H⋯*A*	*D*⋯*A*	*D*—H⋯*A*
O3—H3*A*⋯N3^i^	0.99 (6)	1.86 (6)	2.809 (4)	160 (5)

Symmetry code: (i) 
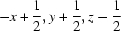
.

**Fig. 1.**
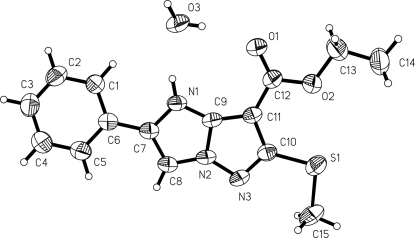
View of the title compound, with displacement ellipsoids drawn at the 40% probability level.

## supplementary crystallographic information

### Comment

Imidazo[1,2-*b*]pyrazole derivatives have been reported to show various biological activities [Vanotti *et al.*, 1994; Kinnamon *et al.*, 2000], in continuation of our research interest in this field [Li *et al.*, 2005], the new title compound has been synthesized in a search for new compounds with better biological activity, and its crystal structure was determined by X-ray diffraction method.

The title compound crystallizes as a monohydrate (Fig. 1), all bond lengths are normal and in a good agreement with those reported previously (Allen *et al.*, 1987). Atoms S1/O1/O2/C12/C13/C14 lie in 1*H*-imidazo[1,2-*b*]pyrazole (C7—C11/N1/N2/N3) plane with maximum least squares plane deviation for C14 0.070 (3) Å. The 1*H*-imidazo[1,2-*b*]pyrazole plane forms dihedral angles of 16.90 (3)° with the benzene ring (C1—C6). π-π stacking interactions (Table 1) are present in the structure. The crystal structure involves intermolecular are O–H···N hydrogen bonds.

### Experimental

A suspended solution in 30 ml of acetonitrile containing 5 mmol (1.01 g) of ethyl 5-amino-3-methylthio-1*H*-pyrazole-4-carboxylate, 5 mmol (1.00) of α-bromoacetophenone and 10 mmol (1.38 g) of sodium carbonate was refluxed for about 10 h until the starting materials were consumed completely, as indicated by TLC. On cooling, the solid was removed through filtration, and the filtrate was evaporated to afford a residue, which was dissolved in 30 ml of absolute ethanol, followed by the addition of several drops of concentrated hydrochloric acid. The resulting solution was then refluxed for about 3 h, and cooled to room temperature. The solution was then evaporated in vacuo to afford the crude product, which was purified by column chromatography using ethyl acetate/petroleum ether (1:5) to give 0.87 g of pure title compound. Crystals suitable for X-ray diffraction were obtained from slow evaporation of a solution of the title compound in dichloromethane/ethyl acetate/petroleum ether (1/2/1) at room temperature.

### Refinement

All H atoms were found on difference maps. The water H atoms were refined freely, giving 0.85 (6) and 0.99 (6)Å, and included in the final cycles of refinement using a riding model, with *U*_iso_(H) = 1.2*U*_eq_(C, N) and 1.5*U*_eq_(C) for the methyl H atoms.

### Crystal data

**Table:**

C_15_H_15_N_3_O_2_S·H_2_O	*F*(000) = 672
*M**_r_* = 319.38	*D*_x_ = 1.335 Mg m^−^^3^
Orthorhombic, *P**n**a*2_1_	Mo *K*α radiation, λ = 0.71073 Å
Hall symbol: P 2c -2n	Cell parameters from 1077 reflections
*a* = 19.017 (2) Å	θ = 2.5–18.0°
*b* = 5.4854 (7) Å	µ = 0.22 mm^−^^1^
*c* = 15.2314 (18) Å	*T* = 273 K
*V* = 1588.9 (3) Å^3^	Block, colorless
*Z* = 4	0.20 × 0.10 × 0.10 mm

### Data collection

**Table:**

Bruker SMART CCD area-detector diffractometer	3326 independent reflections
Radiation source: fine-focus sealed tube	1877 reflections with *I* > 2σ(*I*)
graphite	*R*_int_ = 0.051
φ and ω scans	θ_max_ = 27.5°, θ_min_ = 2.1°
Absorption correction: multi-scan (*SADABS*; Sheldrick, 1996)	*h* = −24→17
*T*_min_ = 0.957, *T*_max_ = 0.978	*k* = −7→6
8763 measured reflections	*l* = −19→19

### Refinement

**Table:**

Refinement on *F*^2^	Secondary atom site location: difference Fourier map
Least-squares matrix: full	Hydrogen site location: inferred from neighbouring sites
*R*[*F*^2^ > 2σ(*F*^2^)] = 0.044	H atoms treated by a mixture of independent and constrained refinement
*wR*(*F*^2^) = 0.094	*w* = 1/[σ^2^(*F*_o_^2^) + (0.0345*P*)^2^] where *P* = (*F*_o_^2^ + 2*F*_c_^2^)/3
*S* = 0.97	(Δ/σ)_max_ = 0.001
3326 reflections	Δρ_max_ = 0.15 e Å^−^^3^
207 parameters	Δρ_min_ = −0.14 e Å^−^^3^
1 restraint	Absolute structure: Flack (1983), 1442 Friedel pairs
Primary atom site location: structure-invariant direct methods	Flack parameter: −0.01 (9)

### Special details

**Table:**

Geometry. All e.s.d.'s (except the e.s.d. in the dihedral angle between two l.s. planes) are estimated using the full covariance matrix. The cell e.s.d.'s are taken into account individually in the estimation of e.s.d.'s in distances, angles and torsion angles; correlations between e.s.d.'s in cell parameters are only used when they are defined by crystal symmetry. An approximate (isotropic) treatment of cell e.s.d.'s is used for estimating e.s.d.'s involving l.s. planes.
Refinement. Refinement of *F*^2^ against ALL reflections. The weighted *R*-factor *wR* and goodness of fit *S* are based on *F*^2^, conventional *R*-factors *R* are based on *F*, with *F* set to zero for negative *F*^2^. The threshold expression of *F*^2^ > σ(*F*^2^) is used only for calculating *R*-factors(gt) *etc*. and is not relevant to the choice of reflections for refinement. *R*-factors based on *F*^2^ are statistically about twice as large as those based on *F*, and *R*- factors based on ALL data will be even larger.

### Fractional atomic coordinates and isotropic or equivalent isotropic displacement parameters (Å^2^)

**Table:**

	*x*	*y*	*z*	*U*_iso_*/*U*_eq_	
S1	0.09788 (5)	−0.14517 (15)	0.84062 (6)	0.0691 (3)	
O1	0.15780 (12)	0.2624 (4)	0.58697 (15)	0.0736 (7)	
O2	0.09476 (11)	−0.0245 (4)	0.65679 (14)	0.0650 (6)	
O3	0.2436 (2)	0.6611 (6)	0.55376 (16)	0.0945 (10)	
H3A	0.275 (3)	0.647 (9)	0.502 (4)	0.16 (2)*	
H3C	0.223 (3)	0.523 (10)	0.551 (3)	0.15 (2)*	
N1	0.25532 (13)	0.5824 (4)	0.73178 (16)	0.0526 (7)	
H1A	0.2568	0.6315	0.6782	0.063*	
N2	0.22812 (12)	0.3832 (5)	0.84912 (16)	0.0525 (6)	
N3	0.19001 (14)	0.2003 (5)	0.88960 (15)	0.0577 (7)	
C1	0.34450 (18)	1.0192 (6)	0.7130 (2)	0.0603 (9)	
H1B	0.3120	0.9869	0.6689	0.072*	
C2	0.3920 (2)	1.2072 (6)	0.7024 (2)	0.0719 (11)	
H2A	0.3909	1.3011	0.6516	0.086*	
C3	0.4406 (2)	1.2562 (7)	0.7662 (3)	0.0738 (10)	
H3B	0.4727	1.3824	0.7588	0.089*	
C4	0.4416 (2)	1.1188 (7)	0.8405 (3)	0.0839 (10)	
H4A	0.4748	1.1508	0.8838	0.101*	
C5	0.39376 (19)	0.9320 (6)	0.8522 (3)	0.0759 (10)	
H5A	0.3947	0.8413	0.9038	0.091*	
C6	0.34440 (16)	0.8779 (6)	0.7882 (2)	0.0512 (8)	
C7	0.29409 (17)	0.6804 (5)	0.8010 (2)	0.0527 (8)	
C8	0.27674 (16)	0.5545 (6)	0.8748 (2)	0.0544 (9)	
H8A	0.2942	0.5792	0.9312	0.065*	
C9	0.21516 (17)	0.3985 (6)	0.76236 (19)	0.0490 (8)	
C10	0.15369 (15)	0.1013 (5)	0.8233 (2)	0.0511 (8)	
C11	0.16699 (17)	0.2151 (5)	0.7410 (2)	0.0502 (8)	
C12	0.14107 (16)	0.1594 (6)	0.6547 (2)	0.0542 (8)	
C13	0.0647 (2)	−0.1008 (7)	0.5744 (2)	0.0850 (12)	
H13A	0.1009	−0.1654	0.5360	0.102*	
H13B	0.0422	0.0358	0.5452	0.102*	
C14	0.0120 (2)	−0.2929 (7)	0.5953 (3)	0.0986 (13)	
H14A	−0.0095	−0.3494	0.5421	0.148*	
H14B	−0.0235	−0.2265	0.6333	0.148*	
H14C	0.0349	−0.4267	0.6242	0.148*	
C15	0.1101 (2)	−0.1931 (7)	0.9562 (3)	0.0873 (13)	
H15A	0.0816	−0.3276	0.9751	0.131*	
H15B	0.0966	−0.0488	0.9876	0.131*	
H15C	0.1587	−0.2285	0.9677	0.131*	

### Atomic displacement parameters (Å^2^)

**Table:**

	*U*^11^	*U*^22^	*U*^33^	*U*^12^	*U*^13^	*U*^23^
S1	0.0711 (6)	0.0678 (5)	0.0682 (6)	−0.0051 (5)	0.0093 (5)	0.0161 (5)
O1	0.0939 (19)	0.0829 (16)	0.0440 (12)	−0.0255 (15)	−0.0010 (14)	0.0096 (12)
O2	0.0703 (14)	0.0722 (15)	0.0526 (14)	−0.0146 (14)	−0.0035 (12)	0.0025 (12)
O3	0.131 (3)	0.107 (3)	0.0453 (15)	−0.041 (2)	0.0042 (15)	0.0143 (15)
N1	0.0628 (17)	0.0571 (16)	0.0377 (15)	0.0017 (14)	−0.0024 (13)	0.0054 (14)
N2	0.0642 (17)	0.0573 (16)	0.0359 (15)	0.0048 (13)	−0.0019 (14)	0.0087 (13)
N3	0.0700 (19)	0.0583 (16)	0.0448 (16)	−0.0010 (15)	0.0068 (15)	0.0150 (14)
C1	0.057 (2)	0.067 (2)	0.056 (2)	−0.0041 (19)	−0.0011 (16)	0.0014 (18)
C2	0.082 (3)	0.074 (3)	0.060 (2)	−0.006 (2)	0.013 (2)	0.007 (2)
C3	0.070 (2)	0.076 (2)	0.076 (3)	−0.015 (2)	0.010 (2)	−0.010 (2)
C4	0.089 (3)	0.088 (3)	0.075 (3)	−0.019 (2)	−0.015 (3)	−0.003 (3)
C5	0.088 (3)	0.077 (2)	0.062 (2)	−0.011 (2)	−0.018 (2)	0.006 (2)
C6	0.054 (2)	0.0534 (18)	0.046 (2)	0.0094 (16)	0.0014 (15)	−0.0006 (15)
C7	0.060 (2)	0.0549 (19)	0.0435 (19)	0.0042 (17)	−0.0012 (17)	0.0009 (17)
C8	0.062 (2)	0.060 (2)	0.0403 (18)	0.0040 (18)	−0.0048 (15)	0.0020 (16)
C9	0.0551 (19)	0.056 (2)	0.0365 (18)	0.0079 (17)	0.0028 (15)	0.0095 (16)
C10	0.0534 (19)	0.0508 (18)	0.049 (2)	0.0074 (15)	0.0022 (15)	0.0050 (16)
C11	0.057 (2)	0.054 (2)	0.0405 (17)	0.0023 (17)	0.0057 (16)	0.0049 (16)
C12	0.055 (2)	0.055 (2)	0.053 (2)	0.0005 (17)	0.0055 (17)	0.0062 (18)
C13	0.087 (3)	0.101 (3)	0.066 (3)	−0.026 (2)	−0.005 (2)	−0.011 (2)
C14	0.108 (3)	0.094 (3)	0.095 (3)	−0.043 (3)	0.001 (3)	−0.013 (2)
C15	0.107 (3)	0.089 (3)	0.067 (3)	−0.005 (2)	0.026 (2)	0.027 (2)

### Geometric parameters (Å, °)

**Table:**

S1—C10	1.739 (3)	C3—H3B	0.9300
S1—C15	1.795 (4)	C4—C5	1.382 (4)
O1—C12	1.219 (4)	C4—H4A	0.9300
O2—C12	1.339 (3)	C5—C6	1.385 (4)
O2—C13	1.442 (4)	C5—H5A	0.9300
O3—H3A	0.99 (6)	C6—C7	1.459 (4)
O3—H3C	0.85 (6)	C7—C8	1.359 (4)
N1—C9	1.348 (3)	C8—H8A	0.9300
N1—C7	1.395 (4)	C9—C11	1.399 (4)
N1—H1A	0.8600	C10—C11	1.423 (4)
N2—C9	1.347 (4)	C11—C12	1.436 (4)
N2—C8	1.375 (4)	C13—C14	1.489 (5)
N2—N3	1.383 (3)	C13—H13A	0.9700
N3—C10	1.339 (4)	C13—H13B	0.9700
C1—C2	1.380 (4)	C14—H14A	0.9600
C1—C6	1.383 (4)	C14—H14B	0.9600
C1—H1B	0.9300	C14—H14C	0.9600
C2—C3	1.368 (5)	C15—H15A	0.9600
C2—H2A	0.9300	C15—H15B	0.9600
C3—C4	1.360 (5)	C15—H15C	0.9600
			
Cg1···Cg2^i^	3.643 (2)		
			
C10—S1—C15	100.60 (17)	C7—C8—H8A	127.0
C12—O2—C13	117.3 (3)	N2—C8—H8A	127.0
H3A—O3—H3C	99 (4)	N2—C9—N1	106.4 (3)
C9—N1—C7	109.0 (3)	N2—C9—C11	107.7 (3)
C9—N1—H1A	125.5	N1—C9—C11	146.0 (3)
C7—N1—H1A	125.5	N3—C10—C11	113.3 (3)
C9—N2—C8	111.1 (3)	N3—C10—S1	121.0 (2)
C9—N2—N3	112.8 (3)	C11—C10—S1	125.7 (2)
C8—N2—N3	136.2 (3)	C9—C11—C10	103.1 (3)
C10—N3—N2	103.2 (2)	C9—C11—C12	126.2 (3)
C2—C1—C6	121.1 (3)	C10—C11—C12	130.6 (3)
C2—C1—H1B	119.4	O1—C12—O2	122.7 (3)
C6—C1—H1B	119.4	O1—C12—C11	125.9 (3)
C3—C2—C1	120.4 (3)	O2—C12—C11	111.4 (3)
C3—C2—H2A	119.8	O2—C13—C14	106.6 (3)
C1—C2—H2A	119.8	O2—C13—H13A	110.4
C4—C3—C2	119.5 (4)	C14—C13—H13A	110.4
C4—C3—H3B	120.3	O2—C13—H13B	110.4
C2—C3—H3B	120.3	C14—C13—H13B	110.4
C3—C4—C5	120.6 (4)	H13A—C13—H13B	108.6
C3—C4—H4A	119.7	C13—C14—H14A	109.5
C5—C4—H4A	119.7	C13—C14—H14B	109.5
C4—C5—C6	120.9 (4)	H14A—C14—H14B	109.5
C4—C5—H5A	119.5	C13—C14—H14C	109.5
C6—C5—H5A	119.5	H14A—C14—H14C	109.5
C1—C6—C5	117.5 (3)	H14B—C14—H14C	109.5
C1—C6—C7	121.9 (3)	S1—C15—H15A	109.5
C5—C6—C7	120.6 (3)	S1—C15—H15B	109.5
C8—C7—N1	107.5 (3)	H15A—C15—H15B	109.5
C8—C7—C6	130.3 (3)	S1—C15—H15C	109.5
N1—C7—C6	122.1 (3)	H15A—C15—H15C	109.5
C7—C8—N2	106.0 (3)	H15B—C15—H15C	109.5
			
C9—N2—N3—C10	0.9 (3)	N3—N2—C9—C11	−1.2 (3)
C8—N2—N3—C10	−178.6 (3)	C7—N1—C9—N2	0.5 (3)
C6—C1—C2—C3	0.6 (5)	C7—N1—C9—C11	−177.5 (4)
C1—C2—C3—C4	−0.3 (5)	N2—N3—C10—C11	−0.2 (3)
C2—C3—C4—C5	−0.5 (6)	N2—N3—C10—S1	178.80 (19)
C3—C4—C5—C6	1.0 (6)	C15—S1—C10—N3	−0.8 (3)
C2—C1—C6—C5	−0.1 (5)	C15—S1—C10—C11	178.0 (3)
C2—C1—C6—C7	179.2 (3)	N2—C9—C11—C10	1.0 (3)
C4—C5—C6—C1	−0.7 (5)	N1—C9—C11—C10	179.0 (4)
C4—C5—C6—C7	179.9 (3)	N2—C9—C11—C12	−177.1 (3)
C9—N1—C7—C8	−0.4 (3)	N1—C9—C11—C12	0.9 (7)
C9—N1—C7—C6	178.0 (2)	N3—C10—C11—C9	−0.5 (3)
C1—C6—C7—C8	−165.9 (3)	S1—C10—C11—C9	−179.5 (2)
C5—C6—C7—C8	13.5 (5)	N3—C10—C11—C12	177.5 (3)
C1—C6—C7—N1	16.2 (4)	S1—C10—C11—C12	−1.4 (5)
C5—C6—C7—N1	−164.4 (3)	C13—O2—C12—O1	0.4 (4)
N1—C7—C8—N2	0.1 (3)	C13—O2—C12—C11	−179.7 (3)
C6—C7—C8—N2	−178.1 (3)	C9—C11—C12—O1	0.8 (5)
C9—N2—C8—C7	0.2 (3)	C10—C11—C12—O1	−176.9 (3)
N3—N2—C8—C7	179.7 (3)	C9—C11—C12—O2	−179.1 (3)
C8—N2—C9—N1	−0.5 (3)	C10—C11—C12—O2	3.2 (4)
N3—N2—C9—N1	179.9 (2)	C12—O2—C13—C14	−176.1 (3)
C8—N2—C9—C11	178.4 (2)		

Symmetry codes: (i) *x*, *y*+1, *z*.

### Hydrogen-bond geometry (Å, °)

**Table:**

*D*—H···*A*	*D*—H	H···*A*	*D*···*A*	*D*—H···*A*
O3—H3A···N3^ii^	0.99 (6)	1.86 (6)	2.809 (4)	160 (5)

Symmetry codes: (ii) −*x*+1/2, *y*+1/2, *z*−1/2.
